# Supplementary material for: Geography of Indian Butterflies: Patterns Revealed by Checklists of Federal States
Source: Insects. 2023 Jun 13;14(6):549. doi: 10.3390/insects14060549 (PMC10299651; doi:10.3390/insects14060549)
Supplement: Supplementary file 1 [file insects-14-00549-s001.zip › Sup F1.pdf]

Supplementary figures S1: Detailed results of the PCA analysis of 19 bioclimatic variables (Karger et al. 2017), used to obtain the composite climatic variables Clim1–4

Eigenvalues of the analysis were 0.336, 0.197, 0.135, 0.075

Panel 1: PCA biplot of the first (Clim1) and second (Clim2) ordination axes.

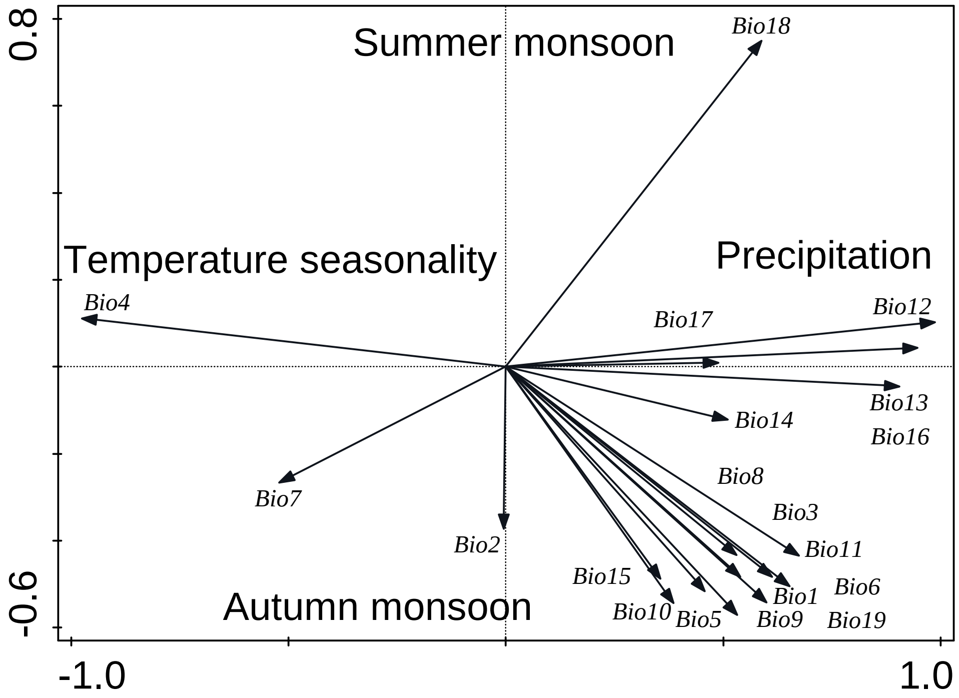

Panel 2: PCA biplot of the first (Clim1) and third (Clim3) ordination axes.

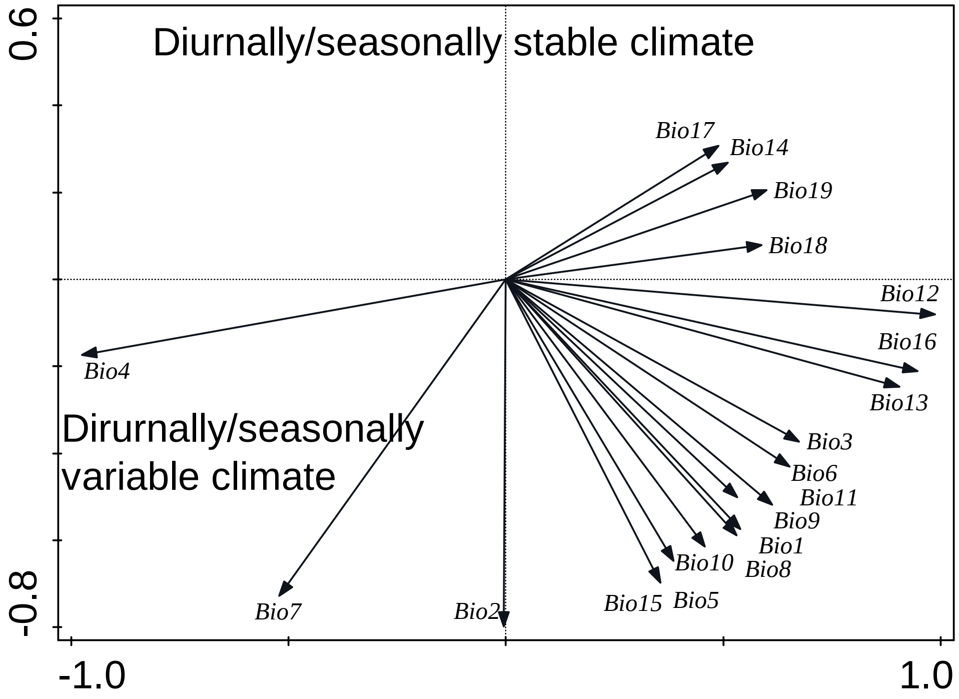

Panel 3: PCA biplot of the first (Clim1) and fourth (Clim4) ordination axes.

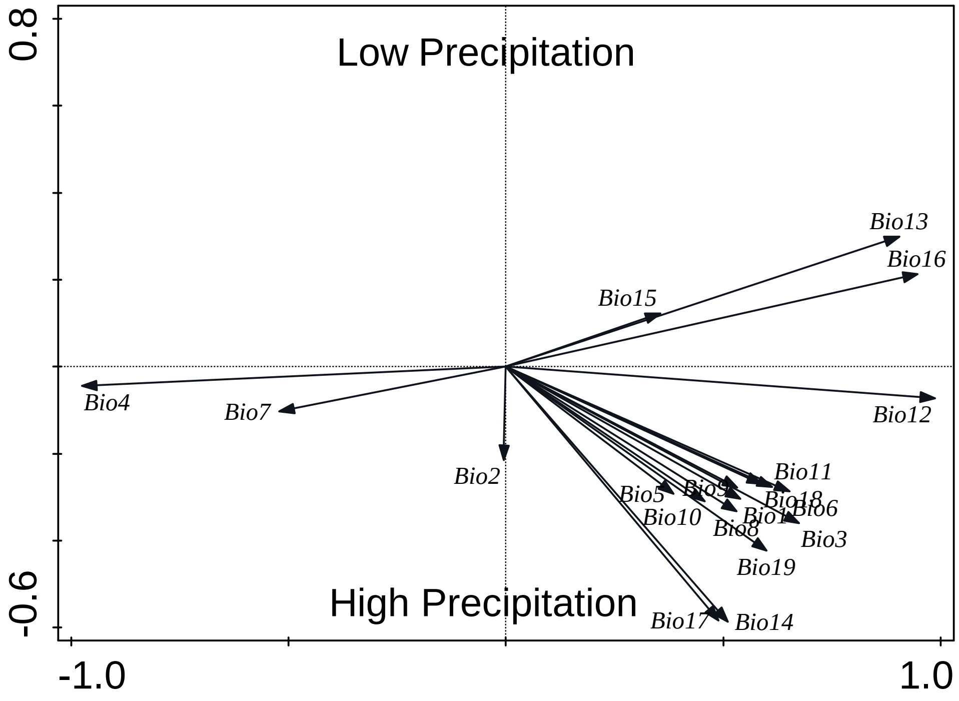

Karger, D.N.; Conrad, O.; Böhner, J.; Kawohl, T.; Kreft, H.; Soria-Auza, R.W.; Zimmermann, N.E.; Linder, H.P.; Kessler, M. 702 Climatologies at high resolution for the earth's land surface areas. Sci. Data 2017, 4, 170122. 703 https://doi.org/10.1038/sdata.2017.122
