# Supplementary figures and images for: Geography of Indian Butterflies: Patterns Revealed by Checklists of Federal States
Source: Insects. 2023 Jun 13;14(6):549. doi: 10.3390/insects14060549 (PMC10299651; doi:10.3390/insects14060549)

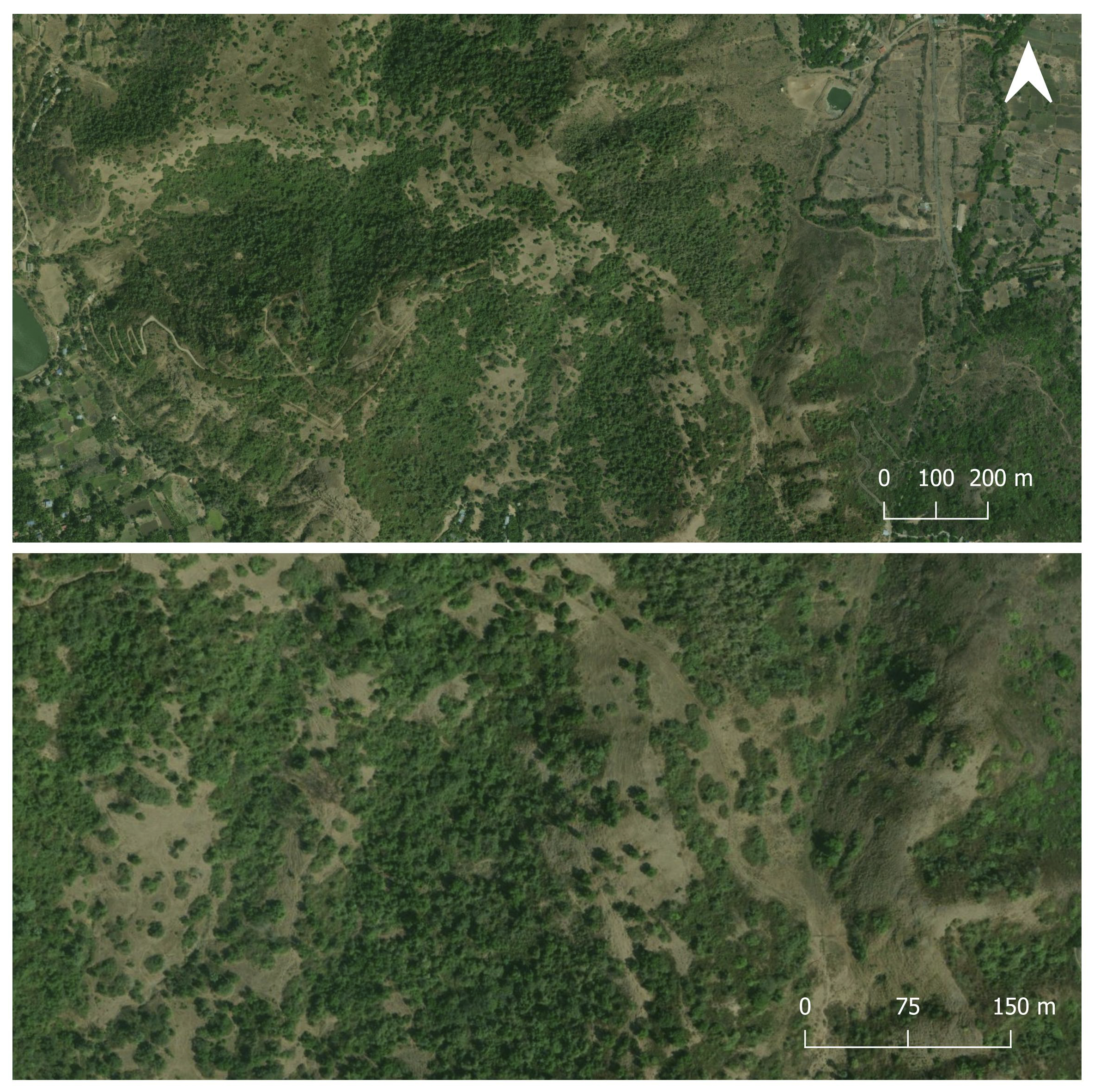

Supplement: Supplementary file 1 [file insects-14-00549-s001.zip › Sup F2.png]
